# Anisotropy dependence of the fluctuation spectroscopy in the critical and gaussian regimes in superconducting NaFe_1−*x*_Co_*x*_As single crystals

**DOI:** 10.1038/s41598-018-26939-8

**Published:** 2018-06-04

**Authors:** D. Ahmad, W. J. Choi, D. Sóñora, Yoon Seok Oh, J. Mosqueira, Tuson Park, Yong Seung Kwon

**Affiliations:** 1 Department of Emerging Materials Science, DGIST, Daegu, 42988 Republic of Korea; 2 Department of Physics, Ulsan National Institute of Science and Technology, Ulsan, 44919 Republic of Korea; 3 LBTS, Departamento de Física de Partículas, Universidade de Santiago de Compostela, E-15782 Santiago de Compostela, Spain; 4 Creative Research Center for Quantum Materials and Superconductivity, Sungkyunkwan University, Suwon, Republic of Korea

## Abstract

We investigate thermal fluctuations in terms of diamagnetism and magnetotransport in superconducting NaFe_1−*x*_Co_*x*_As single crystals with different doping levels. Results show that in the case of optimal doped and lightly overdoped (*x* = 0.03, 0.05) crystals the analysis in the critical as well as in the Gaussian fluctuation regions is consistent with the Ginzburg-Landau 3D fluctuation theory. However, in the case of strongly overdoped samples (*x* ≥ 0.07) the Ullah-Dorsey scaling of the fluctuation induced magnetoconductivity in the critical region confirms that thermal fluctuations exhibit a 3D anisotropic nature only in a narrow temperature region around *T*_*c*_(*H*). This is consistent with the fact that in these samples the fluctuation effects in the Gaussian region above *T*_c_ may be described by the Lawrence-Doniach approach. Our results indicate that the anisotropy of these materials increases significantly with the doping level.

## Introduction

The phenomenological description of preformed Cooper pairs above *T*_*c*_ as a result of fluctuations of the superconducting order parameter has remained as one of the most important topics in the field of superconductivity. In addition to their intrinsic interest, the analysis of fluctuation effects in the vicinity of the transition temperature *T*_*c*_ also allow to obtain superconducting parameters such as the upper critical field, the coherence length, the anisotropy and the dimensionality. One striking feature of the superconducting fluctuations is their effect on vortex motion which in turn results in a rounding effect near *T*_*c*_ in the magneto-resistance and the magnetization^1–5^. The rounding effect is quantified by the so-called Ginzburg number ${{\rm{G}}}_{i}={({k}_{B}/4\pi {\xi }_{ab}(0){\xi }_{c}(0){\rm{\Delta }}c)}^{2}/2$, where ${\rm{\Delta }}c$ is the specific heat jump at *T*_*c*_, ${k}_{B}$ the Boltzmann constant, and ${\xi }_{ab}(0)$ and ${\xi }_{c}(0)$ are the in-plane and *c*-axis coherence lengths extrapolated to 0 K. *G*_*i*_ in the iron-based superconductors was found to be in between the values corresponding to high *T*_*c*_ and conventional low *T*_*c*_ superconductors^6–8^. For example, in SmFeAs_0.85_F_0.15_ a value of ${G}_{i}$ as large as ~1.6 × 10^−2^ was estimated^6,7^, while in Co-doped BaFe_2_As_2_ single crystals ${G}_{i}$ is in the range of ~10^−5^^8^. The value of ${G}_{i}$ in NaFe_1−*x*_Co_*x*_As was estimated to be of the order of 10^−4^ from the ${\xi }_{ab}(0)$ and ${\xi }_{c}(0)$ values obtained below, and from the Δ*c* value in ref.^9^. So far, experimental investigation of the fluctuation effects has been performed through observables such as the specific heat^6,10,11^, the magnetization^11–30^, the electrical conductivity^7,8,11,21–29^ and the microwave conductivity^30^. The fluctuation effects in high *T*_*c*_ superconductors have been well understood in terms of the Lawrence-Doniach (LD) model for layered superconductors^31^. In the case of iron pnictides there’s some controversy about the dimensionality of fluctuation effects. For instance, some works reported a two-dimensional (2D) behavior in compounds from the 1111 family like SmFeAsO^7^, and from the 111 family like LiFeAs^23,24^. However, some recent reports showed a 3D anisotropic behavior in compounds from the same families^6,16,32^.

In this paper, we investigate the superconducting fluctuation effects in the magnetization and electrical conductivity of NaFe_1−*x*_Co_*x*_As single crystals with *x* = 0.03, 0.05, 0.07, 0.073, which cover from the optimal doping to the highly overdoped regime. This compound presents a PbClF-type crystal structure, in which Na^+^-ions are sandwiched between the FeAs layers^33^. The bulk superconductivity is induced upon Co doping, the maximum *T*_*c*_ occurring for *x* = 0.028^9^. We study both the critical and the Gaussian fluctuation regimes, by using the Ullah and Dorsey scaling and, respectively, the 3DGinzburg-Landau approach and the quasi-2D Lawrence-Doniach model^2,26^. This work extends a previous study of critical fluctuation effects in the magnetization of optimally doped NaFe_1−*x*_Co_*x*_As (*x* = 0.03)^16^, and will allow to explore the dependence of the superconducting parameters and of the dimensionality with the doping level.

## Results and Discussion

**Figure 1:**
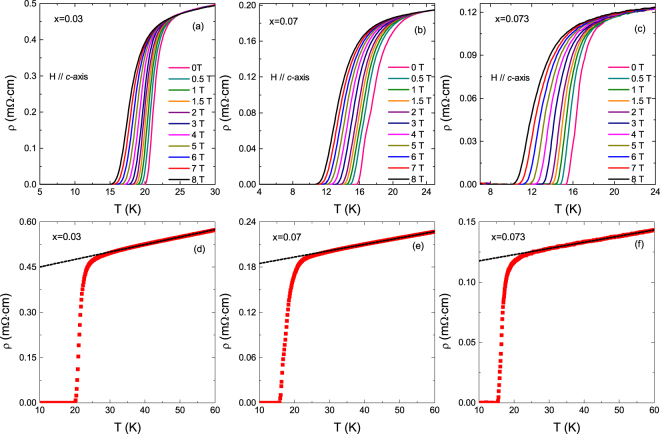
(**a**–**c**) Temperature dependence of resistivity near *T*_c_ for NaFe_1−*x*_Co_*x*_As (*x* = 0.03, 0.07, 0.073) crystals under different magnetic fields up to 8 T applied perpendicular to the crystals’ *ab-*planes. (**d**–**f**) Example (for *µ*_0_*H* = 0 T) of the procedure used to determine the background contribution by a linear fit above 1.5 *T*_c_ (lines).

Figure 1(a–c) presents the temperature dependence of the resistivity near *T*_*c*_ under various magnetic fields applied parallel to the crystals’ *c*-axis in optimally doped (*x* = 0.03) and overdoped (*x* = 0.07, 0.073) NaFe_1−*x*_Co_*x*_As crystals. In zero applied magnetic field, the critical temperatures for *x* = 0.03, 0.07 and 0.073 are 20.9 K, 16.4 K, and 16.3 K, respectively, as determined from the maximum of d*ρ*/d*T*. In this representation it may be already appreciated a rounding just above *T*_*c*_ that increases with the applied magnetic field and that may be attributed to superconducting fluctuations. In Fig. 1(d–f) it is presented an example of the normal-state background extraction procedure, that consist in a linear fit above 1.5 *T*_*c*_, a temperature above which fluctuation effects are expected to be negligible^14,20,26^.

**Figure 2:**
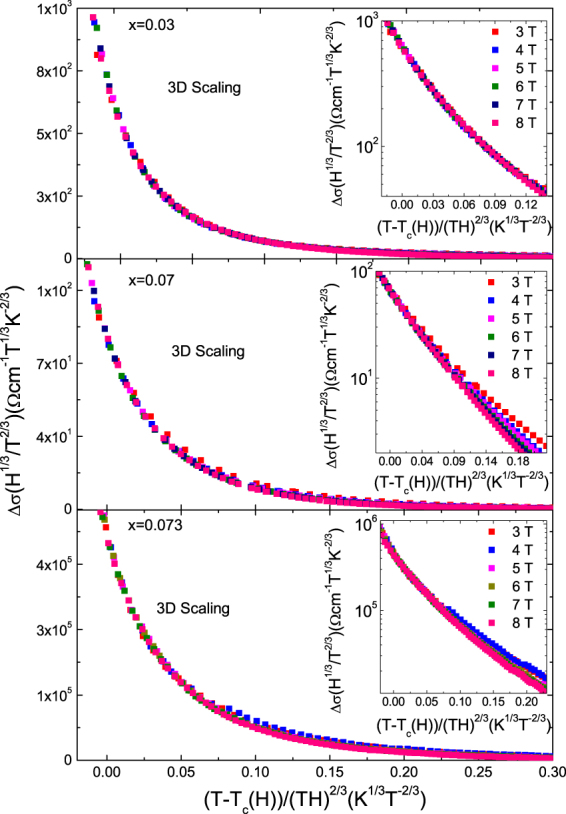
3D LLL scaling of the fluctuation conductivity for NaFe_1−*x*_Co_*x*_As with *x* = 0.03 (**a**), x = 0.07 (**b**), x = 0.073 (**c**). The Insets show the same data in semi-logarithmic scale.

**Figure 3:**
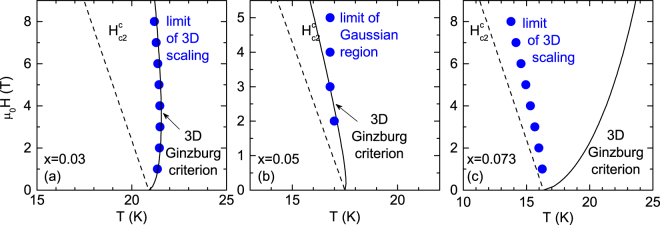
*H*-*T* phase diagrams of NaFe_1−*x*_Co_*x*_As for *H* ⊥*ab*; (**a**) *x* = 0.03, (**b**) *x* = 0.05, and (**c**) *x* = 0.073, respectively. The corresponding *H*_*c*2_(*T*) lines were obtained from the superconducting parameters obtained in the analysis. The symbols are the limit of 3D scaling (*x* = 0.03, 0.073) and limit of Gaussian region (*x* = 0.05). The solid lines are the 3D Ginzburg criterion.

We first analyze fluctuation effects in the critical region around the *T*_*c*_(*H*) line. In this region, in presence of large magnetic fields the paired quasi-particles are limited to remain in their lowest Landau level and the superconducting fluctuations present a one-dimensional character along the magnetic field direction^2,3^. This lower dimensionality significantly enhances the fluctuation effects in a region bounded by the so-called H-dependent Ginzburg criterion, which for 3D materials may be expressed as^6^

1 $$|\frac{T-{T}_{c}(H)}{{T}_{c}}|\le {(\frac{4\pi {k}_{B}{\mu }_{0}H}{{\rm{\Delta }}c{\xi }_{c}(0){\varphi }_{0}})}^{2/3}$$

where ${\varphi }_{0}\,$is the flux quantum and *μ*_0_ the vacuum magnetic permeability. In this region, Ullah and Dorsey (UD) used a self-consistent Hartree approximation to treat the quadratic terms in the Ginzburg-Landau free energy, and obtained an expression for different fluctuation-induced observables. In the case of 3D superconductors, they found that the electrical conductivity follows a scaling behavior that is given by

2 $${\rm{\Delta }}{\sigma }_{3D}^{UD}(T,H)={[\frac{{T}^{2}}{H}]}^{2/3}{f}_{3D}[\frac{T-{T}_{c}(H)}{{(TH)}^{2/3}}]$$

where *f*_3D_ is the scaling function. Figure 2 shows the 3D-UD scaling of Δ*σ* for the optimally doped (*x* = 0.03) and two overdoped (*x* = 0.07 and 0.073) crystals, under different applied fields. The *H*-dependence of the mean-field critical temperature, *T*_*c*_(*H*), is used as a free parameter. The result may be affected by some uncertainty (the difficulties associated to scaling analysis of the electrical conductivity are described in detail in ref.^34^), but the result agrees with the values obtained from a 50% criterion on the normal state resistivity within 2% uncertainty. The insets in Fig. 2 show the same data in semi-logarithmic scale. It is clear from these figures that in the optimally doped crystal the 3D scaling is valid up to higher scaled temperatures than in the *x* = 0.07 and 0.073 crystals. This may also be seen in the *H-T* phase diagrams presented in Fig. 3, where the upper temperature limit of the 3D scaling is compared to the Ginzburg criterion, Eq. (1), as evaluated by using the *ξ*_*c*_(0) value obtained in the analysis in the analysis in the Gaussian region (see below), the Δ*c* value for *x* = 0.03 in ref.^9^, and the Δ*c* values for overdoped crystals expected from the Δ*c*/*T*_*c*_ vs *T*_*c*_ correlation in^35^. In the case of the *x* = 0.03 crystal, the region where the 3D scaling is applicable is in excellent agreement with the prediction. The 3D scaling was also previously observed under similar field amplitudes in the fluctuation-induced magnetic susceptibility of a crystal of the same composition^16^, in single crystals of other iron-pnictide families^6,8,12,14,19,20^, and also in high-*T*_*c*_ superconductors like optimally-doped YBa_2_Cu_3_O_*x*_^5,36^. In the case of the *x* = 0.07 and 0.073 crystals the 3D-LLL scaling fails before reaching the Ginzburg criterion. This could indicate that the anisotropy increases with the doping level (as previously reported in 122 compounds^18^), to the point that a 3D-2D transition could appear on increasing the temperature above *T*_*c*_. The analysis in the Gaussian region presented below seems to support this scenario.

From a linear extrapolation to *T* = 0 K of the *T*_*c*_(*H*) data obtained in the scalings we obtained that the upper critical fields *H*_*c*2_(0) for the *x* = 0.03, 0.07, and 0.073 crystals were estimated to be 58 T, 38 T and 41 T, respectively. From these values, the Ginzburg-Landau in-plane coherence length amplitudes, *ξ*_ab_(0) = [ϕ_0_/2π*μ*_0_*H*_*c*2_(0)]^1/2^ resulted to be 2.4 nm, 2.9 nm, and 2.8 nm, respectively. In spite of the uncertainty associated to the extrapolation to *T* = 0 K, these values are in reasonable agreement with the ones listed in Table 1, derived from the subsequent analysis in the Gaussian region (see below).

**Table 1:** Superconducting parameters of the studied NaFe_1−*x*_Co_*x*_As single crystals.

Value of x	*T*_*c*_ (K)	*ξ*_*c*_(0) (nm)	*ξ*_*ab*_(0) (nm)	*μ*_0_*H*_*c*2_(0) (T)	*γ*
0.03	20.9	0.8	2.4	55	3
0.05	17.5	1.9	3.3	30	1.75
0.073	16.3	0.23	3.36	29.1	14.6

**Figure 4:**
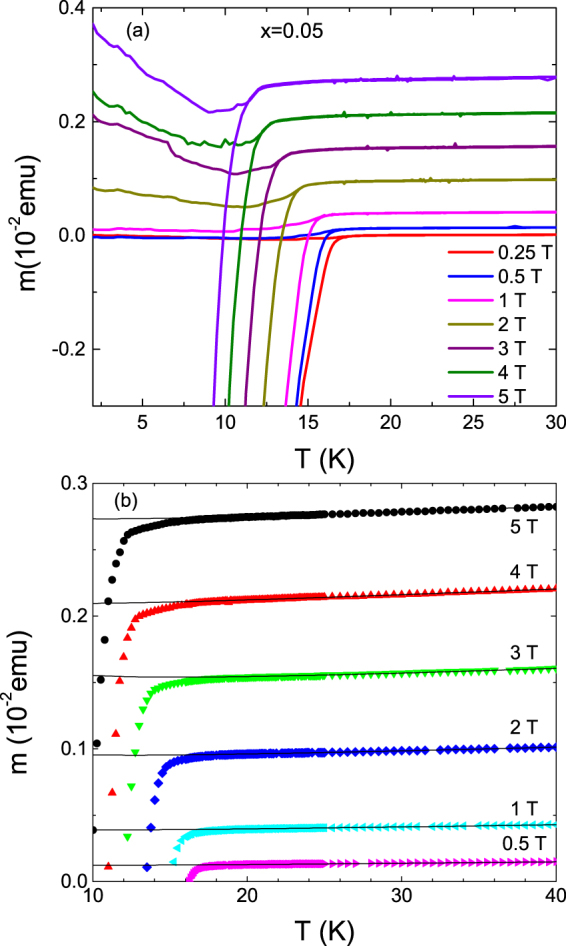
**(a)** Temperature dependence of the magnetic moment of the NaFe_1−*x*_Co_*x*_As (*x* = 0.05) single crystal. These measurements were performed under different magnetic fields applied parallel to *c*-axis in ZFC and FC modes. (**b**) The background contribution (solid lines) were determined by fitting a Curie-like function above 25 K (where fluctuation effects are expected to be negligible), and up to 40 K.

**Figure 5:**
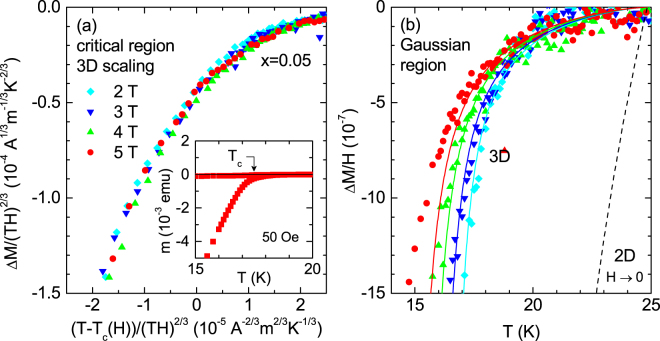
Analysis of the fluctuation magnetization (measured with *H*//*c*) for the NaFe_1−*x*_Co_*x*_As (*x* = 0.05) crystal. (**a**) 3D-UD scaling in the critical region, obtained by using *T*_*c*_ = 17.5 K and *B*_*c*2_(0) = 30 T. (**b**) Temperature dependence of the fluctuation magnetic susceptibility in the Gaussian region. The solid lines correspond to the GL approach for 3D-anisotropic superconductors (Eq. 3), evaluated with the same *T*_*c*_ and *B*_*c*2_(0) values, and with *γ* = 1.75. For comparison, the prediction of the 2D approach is also shown as a dashed line (see the main text for details).

In order to further investigate the fluctuation effects in the critical region, we measured the temperature dependence of the magnetic moment for the slightly overdoped crystal (*x* = 0.05). These measurements, presented in Fig. 4(a), were performed with magnetic fields up to 5 T perpendicular to the ab layers, under zero-field-cooled (ZFC) and field-cooled (FC) conditions. As it may be seen in this figure, a rounding effect just below *T*_*c*_ (which becomes more prominent upon increasing the applied magnetic field) and a broadening of the reversible region (where the ZFC and FC curves coincide) below *T*_*c*_, are substantial evidences of the fluctuation effects in the critical region, in agreement with recent results^13,14,16^. In this case the background contribution (which is mainly due to the crystal’s normal state) was determined by fitting a curie-like dependence *m*_B_(*T*) = *a* + b*T* + *c*/*T* to the as-measured *m*(*T*)_*H*_ data in the temperature range from 25 K to 45 K (solid lines in Fig. 4(b)). The 3D-UD scaling of the fluctuation magnetization in the critical region is presented in Fig. 5(a). In this case the scaling variables are [*T* − *T*_c_(*H*)]/(*TH*)^2/3^ for the temperature and *M*/(*TH*)^2/3^ for the magnetization. We assumed a linear *T*_*c*_(*H*) behavior and obtained a good scaling with *T*_*c*_ = 17.5 K and *μ*_0_*H*_*c*2_(0) = 30 T, see Fig. 5(a). These values are consistent with the analysis in the Gaussian region presented below (see Table 1).

In what follows we complement our study with the analysis of fluctuation effects in the Gaussian region well above the *T*_*c*_(*H*) line, where the quartic term in the free energy may be neglected. Contrary to the critical region, where the different observables present a relatively smooth temperature dependence, in the Gaussian region they present a divergent behavior on approaching the superconducting transition, and this requires that the superconducting transition width, Δ*T*_*c*_, is small relative to the *T*_*c*_ value. In particular, it is expected that a possible *T*_*c*_ distribution affects the Gaussian fluctuation effects below a temperature roughly given by *T*_*inh*_ ~ *T*_*c*_ + Δ*T*_*c*_. In the presence of a magnetic field, due to the *T*_c_(*H*) shift, the region affected by the *T*_c_ distribution is displaced to lower temperatures according to *T*_*inh*_ ~ *T*_c_ + Δ*T*_*c*_ − *H*/[*H*_*c*2_(0)/*T*_c_]. In the case of the *x* = 0.05 crystal Δ*T*_*c*_ is estimated to be ~1 K from the temperature above *T*_*c*_ at which the low-field magnetic moment (inset in Fig. 5) becomes 10^−3^ times the low-temperature saturation value. By taking into account the *μ*_0_*H*_*c*2_(0) value for this sample (30 T), it is expected that a 2 T magnetic field is enough to displace *T*_*inh*_ below the own *T*_*c*_ value. In Fig. 5(b) we present the analysis of the fluctuation magnetization in the Gaussian region in terms of the Ginzburg-Landau approach for three-dimensional anisotropic superconductors under finite applied magnetic fields, which may be expressed as^37^

3 $$\begin{array}{rcl}{M}_{fl}^{\parallel }(T,H) & = & -\,\frac{{K}_{B}T\gamma }{\pi {\varphi }_{0}{\xi }_{ab}(0)}{\int }_{0}^{\sqrt{c-\varepsilon }}dq\,[\frac{c-\varepsilon }{2h}-\,\mathrm{ln}\,{\rm{\Gamma }}(\frac{\varepsilon +h+{q}^{2}}{2h})\\  &  & +\,(\frac{\varepsilon +{q}^{2}}{2h})\psi (\frac{\varepsilon +h+{q}^{2}}{2h})+\,\mathrm{ln}\,{\rm{\Gamma }}(\frac{c+h+{q}^{2}}{2h})\\  &  & -\,(\frac{c+{q}^{2}}{2h})\psi (\frac{c+h+{q}^{2}}{2h})].\end{array}$$

Here *Γ* and *ψ* are the gamma and digamma functions, respectively, *ε* = ln(*T*/*T*_*c*_) the reduced temperature, $h=\frac{H}{{H}_{c2}(0)}=H/[{\varphi }_{0}/2\pi {\mu }_{0}\,{\xi }_{ab}^{2}(0)]$ the reduced magnetic field, *γ* the anisotropy factor, and *c* the total energy cutoff constant, introduced to take into account short-wavelength effects^38^. To compare Eq. (3) with the data we used the *T*_*c*_ and *μ*_0_*H*_*c*2_(0) values resulting from the scaling in the critical region, and used for the remaining parameters *γ* = 1.75 and *c* = 0.35 (in agreement with the one found in other iron-based superconductors^18,26,27^). A good agreement with the experimental results is obtained, confirming the 3D nature of the *x* = 0.05 crystal. As commented above, the data obtained with magnetic fields below 2 T (not shown) do not follow the Gaussian approach, probably due to a possible *T*_c_ distribution. However, it has been also proposed that under these low field amplitudes phase fluctuations may play an important role^15,26^. Another possibility may be that *H*_*c*2_(*T*) is not linear at these field amplitudes (near *T*_*c*_), an effect that some attribute to the multiband nature of these compounds^19,39,40^. Just for completeness, we also include as a dashed line the prediction of the 2D-GL approach for *H* = 0 (Eq. (7) in ref.^20^ after setting *r* = 0), that strongly overestimates the measured Δ*M*/*H* amplitude. Finally, as it may be seen in Fig. 3(b), the lower temperature limit of applicability of the Gaussian approach is close to the prediction of the *H*-dependent Ginzburg criterion for the onset of critical fluctuation effects, Eq. (1), as evaluated with the *ξ*_*c*_(0) value in Table 1, and the Δ*c* value estimated from ref.^35^.

**Figure 6:**
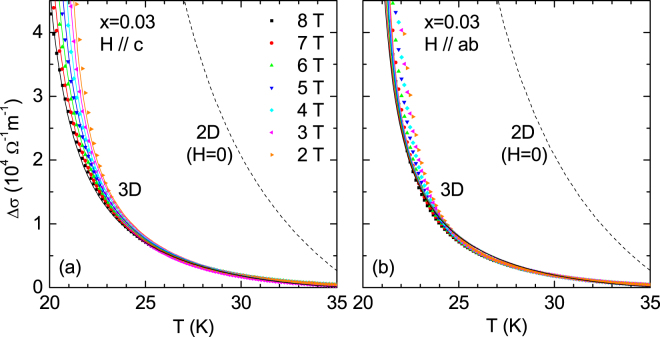
Temperature dependence of the fluctuation conductivity in the Gaussian region for the NaFe_1−*x*_Co_*x*_As (*x* = 0.03) crystal, for both *H*//*c* (**a**) and *H*//*ab* (**b**) under magnetic fields up to 8 T. The solid lines correspond to the Ginzburg-Landau approach for 3D-anisotropic superconductors, Eq. (4). For comparison, the prediction of the 2D approach is also shown as a dashed line (see the main text for details).

**Figure 7:**
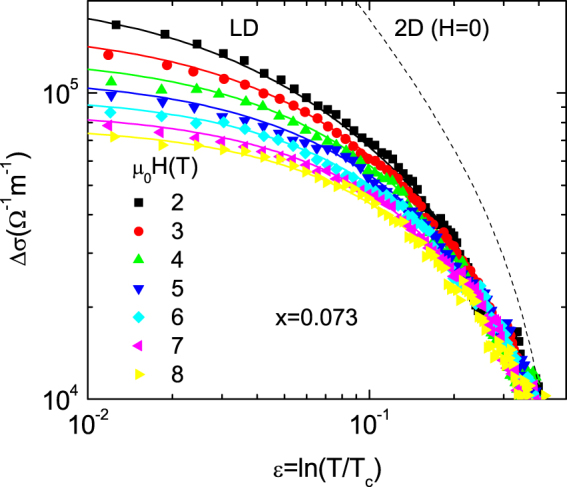
Temperature dependence of the fluctuation conductivity in the Gaussian region for the NaFe_1−*x*_Co_*x*_As (*x* = 0.073) crystal for magnetic fields up to 8 T perpendicular to the *ab* layers. The lines correspond to the Lawrence-Doniach approach for finite applied magnetic fields and under a total-energy cutoff (Eq. 5). For comparison, the prediction of the 2D approach is also shown as a dashed line (see the main text for details).

The analysis of Δ*σ* in the Gaussian region for the optimally doped and highly overdoped crystals is presented in Figs. 6 and 7, respectively. In the case of the *x* = 0.03 and 0.073 crystals, the Δ*T*_*c*_ values (obtained from the FWHM of the d*ρ*/d*T* curve) are about 1.4 K. Taking into account that for these samples *μ*_0_ ${H}_{c2}^{c}(0)$ ∼ 55 T and, respectively, ∼29 T, according to the above argument it is expected that a 2 T field parallel to the *c* axis will allow to analyze fluctuation effects down to ∼0.6 K and ∼0.3 K above *T*_*c*_, respectively. The optimally doped (*x* = 0.03) crystal is analyzed in terms of a GL approach for 3D anisotropic superconductors that includes an energy cutoff to extend the applicability of the GL approach to high reduced temperatures^26^. In the framework of this approach, that has been successfully applied in iron-based superconductors^20,26,27^, the fluctuation-induced conductivity in presence of a magnetic field with an arbitrary orientation is given by

4 $${\rm{\Delta }}{\sigma }_{ab}=\frac{{e}^{2}}{32\hslash \pi {\xi }_{c}(0)}\sqrt{\frac{2}{h}}{\int }_{0}^{\sqrt{\frac{c-\varepsilon }{2h}}}dx[{\psi }^{1}(\frac{\varepsilon +h}{2h}+{x}^{2})-{\psi }^{1}(\frac{c+h}{2h}+{x}^{2})],$$

where $\hslash$ is Planck’s constant and *e* the electron charge. In the absence of applied magnetic fields and also without a cutoff (*c* → ∞), Eq. (4) becomes the well-known Aslamazov–Larkin result^41^. In the case that *H*//*c*, *T*_*c*_ and ${\mu }_{0}{H}_{c2}^{c}(0)$ were estimated to be 20.9 K and, respectively 55 T (i.e., *ξ*_*ab*_(0) = 2.4 nm) from the raw *ρ*(*T*) curves by using a ∼50% criterion, values consistent with the ones resulting from the UD scaling shown in Fig. 2. By using in Eq. (4) these *T*_*c*_ and ${H}_{c2}^{c}(0)$ values and *ξ*_*c*_(0) = 0.8 nm, a rather good agreement is obtained with the experimental data except for the lowest fields (0 and 1 T, not represented in the figure). As commented above this may be due to a possible *T*_*c*_ distribution, although phase fluctuations and the multiband nature of these materials may also play a role^15,19,26,40^. The anisotropy factor resulted to be *γ* = *ξ*_*ab*_(0)/*ξ*_*ab*_(0) ∼3. In case that *H*//*ab*, a relative good agreement was obtained without any free parameter by using a parallel upper critical field of ${\mu }_{0}{H}_{c2}^{ab}(0)=\gamma {\mu }_{0}{H}_{c2}^{c}(0)\,=\,$16  T and to be consistent, the same values of *T*_*c*_ and *ξ*_*c*_(0). The agreement is not good near *T*_*c*_ and under the lowest fields, probably because the large *μ*_0_ ${H}_{c2}^{ab}(0)\,$value extends the region affected by possible *T*_*c*_ distribution up to higher temperatures under the same applied fields (e.g., up to ∼1 K above *T*_*c*_ under a 2 T field parallel to the ab layers). Again, just for completeness, the prediction of the 2D approach for *H* = 0 (that may be obtained from Eq. (4) in ref.^20^ after setting *r* = 0, and that does not depend on any free parameter) is also included. Also in this case it strongly overestimates the observed *Δσ*.

In the case of the overdoped (*x* = 0.073) crystal, we failed to apply the 3D-GL approach for finite fields that was successfully applied in the case of optimal doped and lightly overdoped samples. As the comparison with the UD approach in the critical region suggests a dimensional transition in highly overdoped crystals, we will now probe a quasi-2DLawrence-Doniach (LD) approach for the in-plane fluctuation conductivity valid in presence of finite applied magnetic fields^20^. For magnetic fields perpendicular to the *ab* layers it reads

5 $${\rm{\Delta }}{\sigma }_{LD}(\varepsilon ,h)=\frac{{e}^{2}}{64\pi \hslash }\frac{1}{h}{\int }_{-\pi /s}^{\pi /s}d{k}_{z}[{\psi }^{/}(\frac{{\epsilon }+h+{\omega }_{kz}^{LD}}{2h})+{\psi }^{/}(\frac{c+h+{\omega }_{kz}^{LD}}{2h})],$$

where ${\psi }^{/}$ is the polygamma function, ${\omega }_{kz}^{LD}={B}_{LD}[1-\,\cos ({k}_{z}S)]/2$, ${B}_{LD}={[2{\xi }_{c}(0)/s]}^{2}$ is so called Lawrence-Doniach parameter, *S* is the interlayer distance, and *c* is the total-energy cutoff constant that corresponds to *ε*-value at which Δ*σ* vanishes. To compare with the experimental data, we used *T*_*c*_ = 16.3 K (as determined from the analysis of the critical region), *c* = 0.5 (according to the *ε*-value at which at which fluctuation effects vanish), and *s* = 0.74 nm^42^. As it is shown in Fig. 7, an excellent agreement was found by using for the remaining parameters ${\xi }_{c}(0)$ =  0.23 nm and ${\xi }_{ab}(0)$ = 3.36 nm. The ${\xi }_{ab}(0)$ value is close to the one determined from the analysis in the critical region (2.85 nm) which is a consistency check of our analysis. On the other hand, ${\xi }_{c}(0)$ is significantly smaller than the interlayer distance, which justifies the breakdown of the 3D-anisotropic GL approach and the need to use a LD approach. A similar ${\xi }_{c}(0)$ value is also found in optimally doped YBa_2_Cu_3_O_x_ (also presenting a quasi-2D behavior on increasing the temperature above *T*_*c*_), and in highly anisotropic pnictides like Ca_1−*x*_La_*x*_Fe_1−*y*_Ni_*y*_As_2_^20^, and overdoped BaFe_2−x_Ni_x_As_2_^18^. The ${\xi }_{c}(0)$ value reported in ref.^40^ (1.43 nm) is significantly larger. However, in that work ${H}_{c2}(T)$ was estimated from the shift of resistive transition, a procedure that is highly dependent on the criterion used and that leads to a large uncertainty in the ${H}_{c2}(0)$ value, mainly when $H//ab.$ As for the x = 0.05 and 0.03 crystals, the 2D approach (dashed line) strongly overestimates the observed Δ*σ* amplitude.

**Figure 8:**
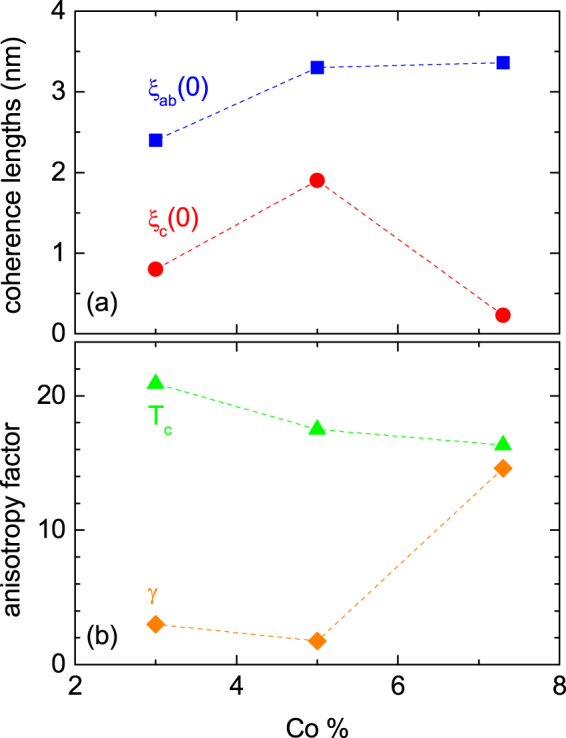
Doping-level dependence of the superconducting parameters, as result from the analysis of the fluctuation effects in the Gaussian region.

The superconducting parameters obtained in the above analysis are listed in Table 1, and represented against the doping level in Fig. 8. The anisotropy factor *γ* in our samples presents a strong dependence on the Co-doping level. While in the optimally-doped sample (*x* = 0.03) *γ* is estimated to be ∼3.6, it is as large as 14.6 in the heavily overdoped sample (it is worth noting that in the slightly overdoped sample (*x* = 0.05) it was found *γ* = 1.75, a value still close to the one found in the optimally-doped compound. However, this result comes from measurements of the fluctuation magnetization, whose amplitude (that is directly proportional to *γ*) may be reduced by an incomplete superconducting volume fraction. This value is even larger than the one observed in 1111 compounds (*γ*(*T*_*c*_) ≈ 6–9)^6,43–45^, and comparable to the one observed in crystals of (Li_1−*x*_Fe_*x*_OH)FeSe and Li_*x*_(NH_3_)_*y*_Fe_2_Se_2_, for which *γ*(*T*_*c*_) ≈ 15^46,47^. The *γ* increase comes essentially from a significant reduction with the doping level of the transverse coherence length (that changes from ∼0.8 nm for *x* = 0.03 to ∼0.23 nm for *x* = 0.07), while *ξ*_*ab*_(0) remains almost constant (it only changes from ∼2.4 nm to ∼3.3 nm). The same effect was also observed in Ba(Fe_1−*x*_Ni_*x*_)_2_As_2_^26^, for which *γ* ∼ 16 was found when *x* = 0.01 (i.e. twice the optimal doping level, as in our present work). In the framework of the LD model *ξ*_*c*_(0) is related to the Josephson coupling constant Γ between adjacent superconducting layers through *ξ*_*c*_(0) = sΓ^1/2^^48,49^. Thus, our results seem to suggest that the FeAs-layers coupling may be significantly weakened by the Co doping.

## Conclusions

We studied the fluctuation effects on the magnetotransport and the magnetization of NaFe_1−*x*_Co_*x*_As (*x* = 0.03, 0.05, 0.07, 0.073) single crystals. The data were compared with the Ullah & Dorsey (UD) scaling approach in the critical region around the *T*_*c*_(*H*) line, and the 3D-GL and quasi-2D LD approaches in the Gaussian region well above *T*_*c*_. The analysis allowed to obtain the dependence with the doping level of fundamental parameters like the coherence lengths and the anisotropy, as well as of the effective dimensionality. While optimally-doped compounds present a moderate anisotropy and a three-dimensional behavior in the critical as well as in Gaussian region, the strongly overdoped compounds are among the most anisotropic iron pnictides, and present 3D characteristics only around *T*_*c*_(*H*), and follow quasi-2D approach well above *T*_*c*_. Our results could be attributed to a weakening of the Josephson coupling between adjacent superconducting FeAs layers, induced by the Co-doping.

## Methods

NaFe_1−*x*_Co_*x*_As (*x* = 0.03, 0.05, 0.07, 0.073) single crystals were grown by using the Bridgman method^22^, by using Na chips, Co chips and FeAs precursors were used as starting materials. The FeAs precursor was synthesized by heating a mixture of Fe and As pieces in an evacuated quartz tube at 500 °C for 48 h, and then heating at 1000 °C for 48 h. In the final step, Na chips, Co chips, and FeAs precursor were mixed in the ratio Na:Fe:Co:As = 2:1-*x*:*x*:1.18 where *x* = 0.03, 0.05, 0.07, 0.073. The mixture for each specific ratio was put in a BN crucible which was in turn put in a W-crucible arc-welded in Ar-atmosphere. Finally, the W-crucible was heated at 1300 °C for about 6 h, followed by a slow downward movement at a rate of 2 mm/h in a vertical Bridgman furnace. After completion of heat treatment, typical dimensions of as-grown single crystals are 0.1 × 1 × 2 mm^3^ for almost each of the three series of samples. We used single crystals from the same batches used in our recent paper^42^. During this study, XRD analyses revealed well defined (0 0 *l*) peaks with FWHM of about 0.05°. Furthermore, SEM images and EDS spectrum revealed that Na, Fe, Co and As are homogeneously distributed. We also performed HR-TEM analysis with FFT images, SAED patterns and theoretical spot diffraction. SAED pattern showed spots only along the zone axis [1 0 0] indicating good crystallinity of the samples. However, some stripes were found because of planar defects. However, at small scale FFT image didn’t reveal any planar defects which indicate, as reported earlier^50^, that these defects may occur due to dual beam FIB and by an intrinsic real structural defect due to Co doping. The in-plane resistivity was measured in the presence of magnetic fields up to 8 T perpendicular and along the crystal’s ab-planes by using a Quantum Design’s Physical Property Measurement System (PPMS). Electrical contacts of ∼1 Ω were prepared by attaching gold wires to the crystals with silver paste in a glove box. The temperature dependence of the magnetization was measured with a commercial SQUID magnetometer (MPMS, Quantum Design). These measurements were performed in both zero-field-cooled (ZFC) and field cooled (FC) modes, with magnetic fields up to 50 kOe.
